# Higher Bioavailability of Calcium in Chickens With a Novel In-Feed Pharmaceutical Formulation

**DOI:** 10.3389/fvets.2020.00343

**Published:** 2020-06-17

**Authors:** Lizbeth Carrillo, María-José Bernad, Minerva Monroy-Barreto, Carlos L. Coello, Héctor Sumano, Lilia Gutiérrez

**Affiliations:** ^1^Departamento de Fisiología y Farmacología, Facultad de Medicina Veterinaria, Universidad Nacional Autónoma de México, Ciudad de México, Mexico; ^2^Departamento de Farmacia, Facultad de Química, Universidad Nacional Autónoma de México, Ciudad de México, Mexico; ^3^Departamento de Química Analítica, Facultad de Química, Universidad Nacional Autónoma de México, Ciudad de México, Mexico; ^4^Departamento de Medicina y Zootecnia de Aves, Facultad de Medicina Veterinaria y Zootecnia, Universidad Nacional Autónoma de México, Ciudad de México, Mexico

**Keywords:** calcium, capsicum, laying hen, relative bioavailability, metabolically-incompetent chicken

## Abstract

Egg production and egg shell quality decrease toward the end of the first laying cycle in hens (approximately by week 80). Even so, farmers often choose to work a second cycle with them. Defective egg shell production has been mainly linked to a decrease in gastrointestinal absorption of calcium. Here we studied pharmaceutically-designed modified-release small pellets (FOLAs) containing calcium to improve calcium bioavailability (F). The influence of FOLA alone or with capsicum-oleoresin was studied in a total of 400 Bovans-White hens randomly divided into four groups of 20 laying hens each and with five replicates per group (*n* = 100) as follows: (1) control group (GC) receiving a diet containing basal levels of 4.1% of calcium-carbonate; (2) group GF treated as GC but with the same dose of calcium-carbonate in FOLA; (3) group GFc5 was treated as GF but with 6 ppm of capsicum-oleoresin (500,000 Scoville Heat Units [SHU]); and (4) group GFc10 treated as GFc5 but with 1,000,000 SHU capsicum-oleoresin. Plasma concentrations of calcium were determined during 5 days at predetermined times sampling more often on days 1 and 5 for blood plasma kinetics of calcium. Relative bioavailability (Fr) values based on the area under the serum calcium concentration vs. time curve (AUC) were obtained and compared to GC. The AUC was statistically different among all groups (*P* < 0.5), but the GFc10 had the greatest Fr (194%), with serum calcium concentrations ranging from 25.37 to 31.2 μg/dL. Calcium residence time (RT) between GC and GF showed no statistical differences while GFc5 and GFc10 had statistically superior RT values. Simultaneously, the number of shell-less eggs per group and their thickness was evaluated by utilizing the same groups but with 150 hens per group on 6 days. Shell-less eggs decreased to zero in Group GFc10 and produced eggs with the greatest shell thickness from day 2 onwards. The inclusion of calcium-carbonate in the pharmaceutical form FOLA induced higher serum calcium concentrations (GF, GFc5, and GFc10) particularly during the night-phase of the hen's cycle—this coincides with the time at which egg shell formation occurs.

## Introduction

The global production of chicken eggs is constantly increasing, e.g., 37.4 million tons of chicken-eggs were produced in 1990, and 80 million tons in 2018. The WHO has estimated that production will have to increase four-fold to meet the global demand for chicken eggs in the next two decades (1, 2). Hence, the poultry industry must be increasingly efficient despite being one of the most efficient and technologically advanced animal production activities (3). Mexico is the first egg consumer in the world with a per-capita intake of 23 kg/year, and a national egg production in 2018 that reached 2,806,000 tons, i.e., the fourth largest egg producer globally (2). Within the national poultry production, ~45% of laying hen flocks are carried to a second production cycle; in other countries, this percentage may be higher than 70% (2). The rationale for avoiding hen replacement is mainly based on costs. While the decrease in production is mainly related to defective egg shell quality, it has been determined that maintaining a second posture cycle is more profitable than replacing the whole flock of laying hens (2, 3).

One of the most important parameters in egg production is egg shell quality. This structure is mainly made of calcium carbonate (CaCO_3_), and there are hardly any egg shell flaws during the first ~35 weeks of age in most laying-hen lines. Thereafter, there is a gradual egg-production drop with decreasing thickness of the shell. This problem has been linked to a decrease in intestinal absorption of calcium of up to 50% on week 40 vs. the initial weeks of egg-production. Hens become genetically impaired to mobilize sufficient calcium when approaching 40 weeks of age—this contributes to defective egg shell formation. The metabolic demand for calcium can lead to osteoporosis when chickens are 38 weeks or older (4, 5).

Most commercial lines of laying hens exhibit a feed consumption rate of ~100 g/hen/day. Their feed is supplemented with 4% total calcium, i.e., 4 g of calcium per day. Considering some variations due to feed handling practices on the farms, most of their food is ingested during the first hours after dawn, and a smaller amount may be eaten during the rest of the day. Of the 4 g of calcium consumed by one hen, ~0.5 g is lost in the feces, about 0.4 g will be excreted in the urine, and 0.1 g will be stored as bone matrix. That leaves ~3 g of Ca available for egg formation: 2 g are deposited as egg shell and the rest is incorporated in the yolk and albumin. During the egg shell calcification process, blood flow through the shell forming-gland in the oviduct increases up to five times (mainly a nocturnal process) (6). Understandably, the calcium requirement of laying hens is 4–6 times greater than that of a non-laying one. The egg is transported into the egg shell forming region in the oviducts. During the last 15 h of egg shell-formation, calcium is incorporated at 100–150 mg/h. Blood calcium ranges from 20 to 30 mg/dL, and 30–40% is from the bone (6, 7).

The intestinal absorption of calcium in the diet is ~40% when the eggshell gland is inactive but reaches 72% when active. This increment mostly occurs at night. Hence, it is important to have higher levels of calcium in the GI tract at this time for proper absorption thus sparing calcium reabsorption from the bones. To promote this, calcium carbonate is usually increased in the diet of hens from week 38 onwards and is usually administered as mixed form with 70% as coarse calcium carbonate (2–4 mm) and 30% as a powder (8, 9). This combination can help calcium be available along the GI tract for longer times. Additionally, the rapid transit time of the GI tract in hens limits the bioavailability (F) of calcium (10) to ~4–5 h per day (11)—this also contributes to the high variability of absorption patterns (12).

Based on these issues, various modified-release feed preparations have been designed to increase the bioavailability of drugs and nutrients (Patent No.MX/a/2012/01,3222 and PCT/MX2013/000137; National Autonomous University of Mexico), and they are collectively referred to as FOLA (F = bioavailability; O = optimum; L = long; A = action) (13, 14). They are small calcium-containing pellets and designed to remain in the GI tract for a longer time to enhance nighttime release of calcium and consequently F. In addition, capsicum-oleoresin can be added to FOLAs to increase the F. Capsicum-oleoresin increases in a concentration-dependant manner, and the amount of divalent and monovalent ions entering the cells containing the VR1 receptor (vanilloid receptor) in intestinal epithelial cells, and there is evidence that the oral administration of capsicum-oleoresin promotes the F of some drugs such as enrofloxacin in poultry (15) and fexofenadine in rats (16). Consequently, we assessed the comparative bioavailability (Fr) of calcium as free calcium-carbonate and as FOLA-pellets with or without capsicum oleoresin. It can also quantify the percent of shell-free eggs in hens already exhibiting this problem.

## Materials and Methods

The study design and animal handling complied with Mexican regulations and ethical standards for the use of experimental animals as laid out by the Universidad National Autonomy de Mexico (UNAM) through the Internal Committee for the Care and Use of Laboratory Animals, and Mexican prescripts in NOM-062-ZOO-1999. It complies with the biosecurity standards of the Poultry Production Research, Extension, and Teaching Center of the Veterinary College (UNAM) (17, 18).

A total of 1,000 85-week-old Bovans-White hens were included. Hens were already into a 10-day forced molt, and had an average weight of 1,520 ± 28 g. Laying hens were allocated in California-type cages, 40 cm wide, 45 cm deep, and 45 cm high for a surface area of 600 cm^2^/hen. They were adapted to an 8–16 h dark/light cycle, had free access to water using cup drinkers at a rate of 3 hens/cup, and their feed was supplied utilizing channel feeders (13.3 cm/hen) following established standards. To avoid extreme/artificial calcium deficiency conditions, the food included a calcium carbonate minimum, i.e., 2% before the commencement of this trial (Table 1) (19).

**Table 1:** Composition and calculated nutrient content in the diet used for 85 weeks-old Bovans-White hens.

**Ingredients**	**(kg)**
Corn	596.170
Soy flour 48%	267.498
Fine calcium carbonate	40.388
Coarse calcium carbonate	60.582
Soy oil	12.506
Orthophosphate 18:20	10.605
Salt	4.072
Vitamin^a^ and mineral^b^ premix	3.00
Mycotoxin sequestrant	2.00
DL-methionine 99%	1.971
Larvadex	0.500
L-Lysine HCl	0.328
Antioxidants	0.150
Fitasa ronozyme (GT)	0.100
Yellow pigment	0.090
Red pigment	0.040
Total	1,000
**Calculated analysis (g/kg)**	
Metabolizable energy (Kcal/kg)	2,859
Crude protein %	18.792
Total calcium%	4.100
Available phosphorus %	0.450
Methionine + Cysteine %	0.800
Lysine %	0.980
Tryptophan %	0.220
Sodium %	0.180

a *Amount/kg: Retinol 0.9 g, cholecalciferol 0.019 g, d-alphatocophero l0.004 g, phylloquinone 1.0 g, riboflavin 4.0 g, cyanocobalamin 0.060 g, pyridoxine 3.0 g, calcium pantothenate 13.0 g, niacin 25 g, biotina 0.063 g, choline chloride 250 g*.

b *Amount/kg: 0.2 g selenium, 0.1 g cobalt, 0.3 g iodine, 10 g copper, 50 g zinc, 100 g iron, 100 g manganese*.

The study was performed in two phases: The calcium kinetics (phase 1) were performed with 400 hens, and the shell-less egg and eggshell thickness study used 600 hens in phase 2. To mimic the calcium-consumption patterns of the hens in productive conditions, the study began on the first day at 6:00 a.m. and was conducted for 5 days in phase 1. In this phase, hens were randomly divided into 4 groups of 100 animals each, and then subdivided into five replicates per group (*n* = 20/replicate). A control group (group GC) was established by supplementing it with 4.1% dietary calcium-carbonate based on the proposed dose-levels for this the lineage and this phase (19). The experimental groups did not receive calcium in their diet—it was incorporated into the manufactured FOLA-pellets as described in the referred patent. Group GF was given 8 g FOLA-pellets/hen containing the 4.1% calcium-carbonate; group GFc5 was provided with 8 g FOLA-pellets/hen also containing 4.1% calcium-carbonate plus 6 ppm of capsicum oleoresin of 500,000 Scoville Heat Units (SHU) (VEPINSA S.A. de C.V., Sinaloa, Mexico), and Group GFc10 was provided with 8 g FOLA-pellets/hen as for GFc5 but with 6 ppm of capsicum oleoresin of 1,000,000 SHU. The feeding time (6 a.m.) was considered time 0 for all groups.

Blood (1–2 mL) was taken from five birds at 0, 1, 2, 4, 6, 8, 10, 12, 14, 16, 18, and 24 h on day 1 and 5; on days 2, 3, and 4, the hens were sampled only at 2, 10, and 18 h. Therefore, each hen was sampled no more than eight times during these 5 days trial in each replicate. This is 25 samples per sampling time in each group. Samples were immediately centrifuged (5,000 g for 15 min), and the serum was recovered for calcium concentration measurements via an Easy Kem Vet® semi-automatic chemical analyzer (Kontrolab, Mexico City). This analytical technique is based on the detection and quantification of a highly specific reaction of calcium with o-cresolftalein-complexone and 8-hydroxyquinoline at pH 8.2. The complex is measured at a wavelength of 570 nm (20). For standardization of the analytical technique, a recovery curve of added calcium chloride (SIGMA) was established in demineralized sterile water with an *r* = 0.99.

A computerized curve stripping program was used to fit and analyze the calcium concentration-vs.-time patterns for each group (PKAnalyst, MicroMath, Salt Lake City, Utah, 1998, USA). Models of best fit (*r*3 = 0.99) were chosen after analysis via the residual sum of squares and the minimal Akaike information criterion. There were several metrics: serum calcium over 24 h (AUC0-24); the maximum serum calcium concentration obtained (CMAX); time in which CMAX (TMAX) is reached; and RT (mean residence time). Statistical differences in serum concentrations of calcium among groups were carried out via a Mann–Whitney *U*-test. The probability values of these data were compared using Kruskal–Wallis tests and Dunnet *t*-tests for independent samples. A statistically significant value was considered when *P* ≤ 0.05. All statistical analyses were performed using SPSS 14.0 for Windows.

Additionally, in phase 2, the number of shell-less eggs produced by 150 hens per group was evaluated, and the thickness of the egg shell (medium, superior, and inferior poles) was measured with a precision digital Vernier calipers with an error of ± 0.01 mm. The egg shell surface was determined via the following formula area = 3.9782 *W* × 0.7056 where *W* is the egg weight (g). The percent productions of shell-less eggs from supplemented hens as in phase 1 were recorded over the following 6 days. These data were statistically compared using the Kruskal–Wallis tests and Dunnet *t*-tests for independence. A statistically significant value was considered when *P* ≤ 0.05. All statistical analyses were performed using SPSS 14.0 for Windows.

## Results

The analytical method used for the evaluation of calcium showed a linearity of 0.001 to 40.0 mg/dL (*r*^2^ = 0.968) with a recovery of 98.6% and a quantification limit of 0.001 mg/dL. The serum concentration of calcium vs. time data was graphed with Origin Lab-Pro-9®. Areas under the concentration vs. time curves 0–24 h, 0–144 h, and 96–120 h were calculated through the trapezoidal method and confirmed with PKAnalyst®.

**Figure 1:**
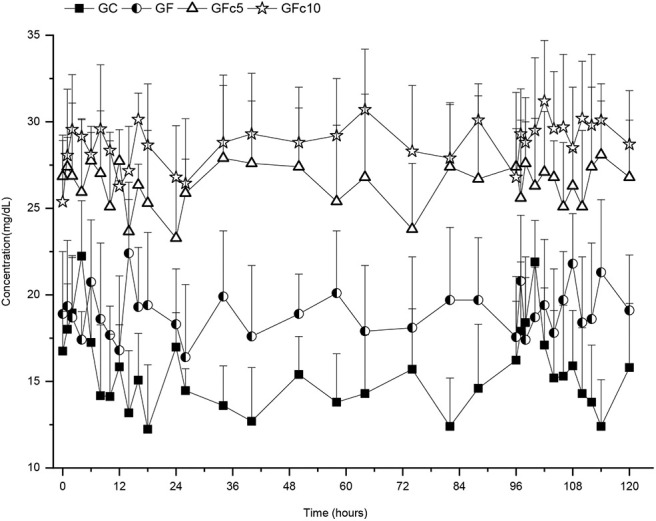
Five days calcium serum concentrations achieved with four calcium sources administered orally in 85-week-old Bovans-White hens: control group (GC), receiving a diet containing basal levels of 4.1% of calcium-carbonate; group GF treated as GC, but including the same dose of calcium-carbonate in FOLA pellets; group GFc5 treated as GF, but adding 6 ppm of capsicum-oleoresin (500,000 Scoville Heat Units [SHU]), and group GFc10 treated as GFc5 but with 1,000,000 SHU capsicum-oleoresin.

**Figure 2:**
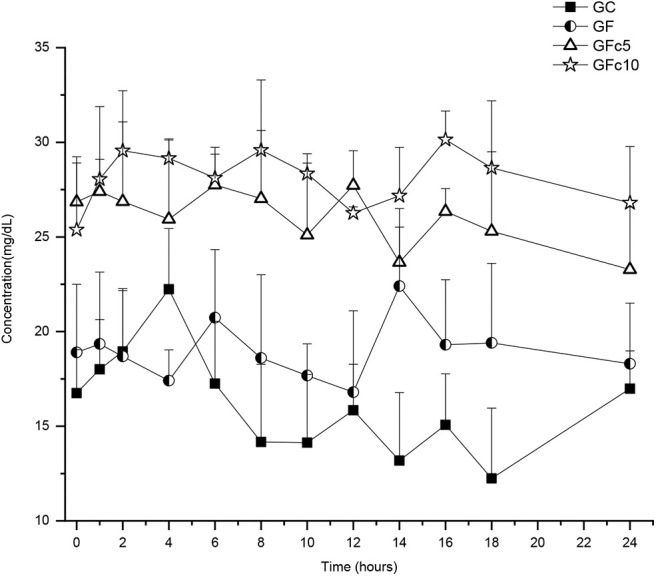
One day calcium serum profiles achieved with four calcium sources administered orally in 85-week-old Bovans-White hens: control group (GC), receiving a diet containing basal levels of 4.1% of calcium-carbonate; group GF treated as GC, but including the same dose of calcium-carbonate in FOLA pellets; group GFc5 treated as GF, but adding 6 ppm of capsicum-oleoresin (500,000 Scoville Heat Units [SHU]), and group GFc10 treated as GFc5 but with 1,000,000 SHU capsicum-oleoresin.

Serum calcium profiles obtained for GC were regarded as basal. Hence, these data were used to calculate relative bioavailability (Fr): AUC_0−24_GF/AUC_0−24_GC × 100. Likewise, Fr values were calculated for the time intervals 24–96 h, 96–120 h, and 0–120 h (Table 2). Values and statistical differences obtained (*P* < 0.5) are listed in Table 2. Serum calcium vs. time profiles are presented in Figures 1, 2. No statistically significant differences were found in any of the kinetic data for serum calcium when comparing data-sets within each group (i.e., calcium concentration ranges, AUC_0−24_ vs. AUC_96−120_; T½α_0−24_ vs. T½α_96−120_; RT_0−24_ vs. RT_96−120_). However, all values obtained among groups were statistically different (*P* < 0.05) except for RT between GC and GF. The GFc10 group had the highest Fr_0−120_ (194%) while the GF obtained had an Fr_0−120_ of only 127%. The addition of capsicum oleoresin in the two SHU potencies significantly increased Fr_0−120_ of calcium (*P* < 0.05) in both groups, i.e., GFc5 and GFc10 (Fr = 177 and 194, respectively). Likewise, the residence times of GFc5 and GFc10 were higher than those obtained in the GC and GF. The GFc10 showed the greatest Ca serum concentrations (*P* < 0.05, for all comparisons), i.e., >31.2 mg/dL while the GC was only 22.2 mg/dL. Predictably, the variables in the GC group were the lowest (*P* < 0.05 for all comparisons). In phase 2, Table 3 shows the percent of shell-less eggs recorded by each group per day. There were no shell-less eggs from day 3 onwards in the GFc10 group. Table 4 shows the mean ± 1SD thickness values of egg shells in the different groups. At day 1, the GF, GFc5, and GFc10 groups showed statistically higher values than GC. From day 2 onwards, GFc10 had the thickest egg shells of all groups (*P* < 0.05).

**Table 2:** Kinetic variables achieved for serum calcium in Bovans-White, second-cycle laying hens, considering four groups (*n* = 20 hens in five replicates): control group (GC), receiving a diet containing basal levels of 4.1% of calcium-carbonate; group GF treated as GC, but including the same dose of calcium-carbonate in FOLA pellets; group GFc5 treated as GF, but adding 6 ppm of capsicum-oleoresin [500,000 Scoville Heat Units (SHU)], and group GFc10 treated as GFc5 but with 1,000,000 SHU capsicum-oleoresin.

**Pharmacokinetic parameter**	**GC**	**GF**	**GFc5**	**GFc10**
	**Mean ± SEM**	**Mean ± SEM**	**Mean ± SEM**	**Mean ± SEM**
AUC_0−24_ (μg/mL*h^−1^)	378 ± 23^a^	455 ± 37^b^	619 ± 85^c^	677 ± 91^d^
Fr_0−24_%	_−_	120	164	179
AUC_96−120_ (μg/mL*h^−1^)	378 ± 21^a^	467 ± 29^b^	642 ± 92^c^	676 ± 84^d^
Fr_96−120_%	_−_	124	170	188
AUC_0−120_ (μg/mL*h^−1^)	1,787 ± 125^a^	2,265 ± 186^b^	3,171 ± 212^c^	3,459 ± 237^d^
Fr_0−120_%	_−_	127	177	194
AUC_24−96_ (μg/mL*h^−1^)	1,031 ± 90^a^	1,343 ± 91^b^	1,910 ± 101^c^	2,072 ± 124^d^
Fr _24−96_%	_−_	130	185	201
T$\frac{1}{2}$_α0−24_ (h)	53 ± 7^a^	83 ± 5^b^	134 ± 9^c^	520 ± 50^d^
T$\frac{1}{2}$_α96−120_ (h)	52 ± 7^a^	8 ± 8^b^	134 ± 11^c^	527 ± 57^d^
AUC_0−∞0−24_	1,403 ± 113^a^	3,254 ± 124^b^	5,287 ± 289^c^	21,115 ± 990^d^
AUC_0−∞96−120_	1,363 ± 102^a^	3,290 ± 121^b^	5,299 ± 224^c^	21,454 ± 921^d^
RT_0−24_ (h)	11 ± 1^a^	12 ± 1^a^	193 ± 11^b^	804 ± 69^c^
RT_96−120_ (h)	11 ± 1^a^	12 ± 2^a^	194 ± 12^b^	806 ± 51^c^
Calcium concentrations range (mg/dl)	12 – 22	16 – 22	23 – 28	25 – 31

*a, b, c, d Different letters within columns indicate a statistically significant difference (P < 0.05). FOLA pellets: F, bioavailability; O, optimum; L, long; A, action. AUC_0−24_; AUC_96−120_; AUC_0−120_; AUC_24−96_, area under the serum calcium-concentration vs. the corresponding time-period. Fr, relative bioavailability, taking GC as basal values; T$\frac{1}{2}$_α0−24_ and T$\frac{1}{2}$_α96−120_, absorption of calcium half-times; AUC_0−∞0−24_ and AUC_0−∞96−120_, values of serum calcium-concentrations vs. time extrapolated to infinitum; RT_0−24_ and RT_96−120_, mean serum residence times for the marked time periods*.

**Table 3:** Percent of shell-less eggs recorded for 6 days with four calcium sources administered orally in 85-week-old Bovans-White hens: control group (GC), receiving a diet containing basal levels of 4.1% of calcium-carbonate; group GF treated as GC, but including the same dose of calcium-carbonate in FOLA pellets; group GFc5 treated as GF, but adding 6 ppm of capsicum-oleoresin (500,000 Scoville Heat Units [SHU]), and group GFc10 treated as GFc5 but with 1,000,000 SHU capsicum-oleoresin.

**Day**	**Percent of shell-less eggs**			
	**GC**	**GF**	**GFc5**	**GFc10**
1	6	5.3	6	6
2	5.3	5.3	4	0.7
3	6	4.7	2.7	0
4	6	3.3	0.7	0
5	5.3	2.7	0	0
6	6	2.7	0	0
±SE	0.019	0.094	0.245	0.219

**Table 4:** Eggshell thickness recorded for 6 days with four calcium sources administered orally in 85-week-old Bovans-White hens: control group (GC), receiving a diet containing basal levels of 4.1% of calcium-carbonate; group GF treated as GC, but including the same dose of calcium-carbonate in FOLA pellets; group GFc5 treated as GF, but adding 6 ppm of capsicum-oleoresin (500,000 Scoville Heat Units [SHU]), and group GFc10 treated as GFc5 but with 1,000,000 SHU capsicum-oleoresin.

**Day**	**Eggshell thickness (mm)**							
	**GC**		**GF**		**GFc5**		**GFc10**	
	**Mean**	**±1 SE**	**Mean**	**±1 SE**	**Mean**	**±1 SE**	**Mean**	**±1 SE**
0	0.34	0.019	0.35	0.01	0.34	0.017	0.35	0.022
1	0.35	0.012	0.37^b^	0.012	0.39^c^	0.013	0.4^c^	0.015
2	0.34	0.024	0.37^b^	0.012	0.4^c^	0.015	0.42^d^	0.016
3	0.35	0.025	0.39^b^	0.011	0.41^c^	0.01	0.45^d^	0.014
4	0.34	0.017	0.4^b^	0.013	0.41^c^	0.012	0.43^d^	0.011
5	0.34	0.019	0.4^b^	0.01	0.42^c^	0.011	0.46^d^	0.013
6	0.35	0.013	0.41^b^	0.011	0.43^c^	0.012	0.46^d^	0.02
SEM	0.018		0.01 1		0.012		0.015	

*b, c, d Different letter in rows indicate statistically significant difference within a day (P < 0.05)*.

## Discussion

The quality of chicken eggs depends on many factors including the bioavailability of calcium. The main source of calcium in hens is calcium carbonate—an economical component utilized in most diets worldwide. However, the absorption capability of calcium by the GI tract of hens is diminished during the second posture cycle (21). This can be overcome by adding more calcium to the hen's diet, i.e., twice the normal level utilized during the first cycle (22, 23). Nonetheless, the absorption of calcium can be low and unpredictable during this second egg-posture cycle (24, 25). This addition can fail to meet the physiological need for calcium. Consequently, defective egg shells appear affecting egg production and profitability during the second egg-laying cycle (26–28). Thus, it is critical to design sources of calcium that can remain available at the GI tract of laying hens and offer good bioavailabilities. In addition, absorption-promoters should be present and linked to the calcium source. The pharmaceutical preparation of FOLA pellets tested here incorporate these features. The composition of FOLA pellets is designed to stay longer in the GI tract for better calcium absorption—particularly at night-time (13, 14) while the addition of capsicum-oleoresin improves bioavailability. Capsicum-oleoresin contains a group of substrates known as capsaicinoids that are responsible for the pungent taste of chili peppers (*Capsicum annuum* and *Capsicum frutescens*). The most relevant active ingredients are capsaicin and dihydrocapsaicin, which represent 80–90% of all capsaicinoids and are found in a 1:1 ratio with small amounts of nordihydrocapsaicin, homocapsaicin, homodihydrocapsaicin, norcapsaicina, nornorcapsaicina, dihydrocapsaicin, homohidrocapsaicin, and nonivamide (29). Neither the precise substance(s) allowing for better F nor how this process occurs are known.

It has been postulated that capsaicin molecules activate the secretion of substance P from nearby nerve terminals, and the capsaicins are inserted into the double lipid layer of cell membranes to modify the selective permeability of the ion channels (30). The blockage of the P-glycoprotein-mediated expulsion pumps in the GI tract cells has also been proposed as a mechanism of action (13, 14); capsaicinoids are known to activate anandamide receptors at the intestinal level thus increasing glucose absorption (31). Capsicum-oleoresin has an antibacterial activity and decreases the fecal release of *Salmonella* sp.; this material is a natural yolk pigment. Thus, capsicum-oleoresin is considered a non-antibiotic growth-promoter and is included in hen diets worldwide at a rate of 0.2% (32). Despite the favorable results obtained for calcium F, the optimal dose ratio of capsicum-oleoresin remains to be determined as well as its possible adverse reactions as a function of inclusion rates. Also, considering the different sources of capsicum and capsaicinoids (33), it is advisable to determine the active chemical fraction(s) responsible for inducing an increase in calcium F.

The outstanding improvement in calcium F using the FOLA pharmaceutical preparation containing capsicum-oleoresin is particularly relevant considering that calcium carbonate absorption can only range from 10 to 14% in these hens. This is explained in terms of its low aqueous solubility because intestinal absorption only occurs with dissolved calcium. Other sources of calcium, i.e., calcium gluconate-lactate possess higher solubility, which in turn may favor absorption up to ~40% (unpublished data). However, this latter source of calcium contains only 12% calcium per gram. Hence, large quantities would be required in the drinking water to meet the calcium demand of laying hens during a second egg-producing cycle. Considering the high costs, an adequate dose delivered through the drinking water appears unlikely.

Finally, the particle size of the calcium source has a direct relationship with its GI transit time and consequently with the dissolution/absorption ratio from this site. Relatively coarse particles (>0.5 cm) show longer transit times in the GI tract and remain available for absorption up to 12 h while finer grains are more easily transported distally but are more readily bioavailable for absorption after feeding (34). For these reasons, calcium carbonate is usually added to the diet of hens in a larger proportion of coarse particles (2–4 mm) and the rest as smaller particles (5, 8, 27, 35, 36). Thus, further studies are needed to define the best proportion of coarse and fine granules of calcium carbonate that should be included in the FOLA-pellets to further optimize calcium absorption in double-cycle egg-laying hens (37).

In conclusion, the calcium requirements for egg production in the second cycle hens can be completely corrected in a few days if calcium is supplemented is in the form of FOLA containing capsicum (particularly 1,000,000 Scoville Heat Units). Large-scale commercial tests in second-cycle hens are needed including full egg quality analysis (low quality eggs may account for up to 50% of the production).

## Data Availability Statement

All datasets presented in this study are included in the article/ supplementary material.

## Ethics Statement

Study design and animal handling complied with Mexican regulations and ethical standards for the usage of experimental animals as laid out by the Universidad National Autonoma de México (UNAM) through the Internal Committee for the Care and Use of Laboratory Animals and Mexican prescripts in NOM-062-ZOO-1999. The data comply with the biosecurity standards of the Poultry Production Research, Extension and Teaching Center of the Veterinary College (UNAM) (17, 18). Written permission was granted in March, 2018.

## Author Contributions

HS and LG conceived and designed the study. These authors together with CC and LC carried out the clinical trial. M-JB and MM-B manufactured and evaluated calcium concentrations. LG and HS carried out the pharmacokinetic and statistical analysis. All authors have read and accepted the manuscript as it is presented to the journal.

## Conflict of Interest

The authors declare that the research was conducted in the absence of any commercial or financial relationships that could be construed as a potential conflict of interest.
